# Facilitators, barriers and support needs for staying at work with a chronic condition: a focus group study

**DOI:** 10.1186/s12889-020-8320-x

**Published:** 2020-02-07

**Authors:** A. R. Bosma, C. R. L. Boot, F. G. Schaafsma, J. R. Anema

**Affiliations:** Department of Public and Occupational Health, Amsterdam Public Health research institute, Amsterdam UMC, VU University Amsterdam, Amsterdam, The Netherlands

**Keywords:** Work, Chronic conditions, Support, Barriers and facilitators, Sustainable employment

## Abstract

**Background:**

Working with a chronic condition can be challenging. Providing support to workers with a chronic condition can help them to stay at work and prevent work-related problems. Workers with a chronic condition who successfully stay at work can provide valuable input for the development of effective supportive interventions to prevent exit from work and facilitate sustainable employment. The aim of this study is to explore the lived experiences of workers with a chronic condition and identify existing barriers, facilitators and possible support needs for staying at work.

**Methods:**

Four focus groups were conducted between August and December 2017 with workers with one or more chronic conditions (*n* = 30). Participants included employees and (partially) self-employed workers. All focus group data were transcribed verbatim and thematically analyzed.

**Results:**

Disclosure and expressing one’s needs were considered important personal facilitators for staying at work. Environmental facilitators included receiving practical information on working with a chronic condition and social and employer support. Environmental barriers were identified in the work environment, the health care system and service provision, e.g., manager and co-worker’s lack of knowledge about working with a chronic condition, a lack of focus on work in the course of treatment for a chronic condition, dissatisfaction with occupational physician support, and the absence of support for self-employed workers. Provided support should be available to all workers, and be proactive and tailored to the workers’ specific support needs.

**Conclusions:**

A variety of facilitators, barriers and support needs were identified in various domains. By addressing environmental barriers (e.g., by integrating work in the course of treatment and creating supportive work environments), sustainable employment by workers with a chronic condition can be promoted.

## Background

Working with a chronic condition can be a struggle, since physical or psychological challenges can hamper work performance, potentially resulting in a loss of productivity, extended or frequent sick leave, or job loss [1–3]. The number of people in the working population with one or more chronic conditions will continue to rise due to a variety of reasons, amongst others an aging population, unhealthy lifestyles and unfavorable working conditions [4, 5]. Although a large percentage of the working population with a chronic condition is able to work, work participation rates among these workers lag behind the general population [6]. Staying at work and prevention of work-related problems among workers with a chronic condition is of significant importance, since return to work after reporting being ill has proven to be difficult [5, 7].

Participation in the workforce positively influences wellbeing and improves quality of life, as it brings purpose to life and fosters socials contacts [8, 9]. Relevant factors enabling workers with a chronic condition to stay at work have been well investigated, and demonstrate that in addition to disease-related factors, personal and environmental factors are critical for sustainable employment [10–12]. A wide variety of interventions have been developed to facilitate sustainable employment for these workers, and are aimed at the work environment (e.g. facilitating work accommodations) or directed at the individual worker (e.g. increasing empowerment and self-management skills) [13–15].

In addition to self-management and empowerment, self-control is a relevant factor for staying at work. Empowerment, self-management and self-control are all concepts that relate to one’s ability to master a life with a chronic condition and maintain quality of life [16–19]. However, some differences between these concepts can be identified. Self-management can, in a broader sense, be defined as the daily management of a chronic condition over the course of the illness, thereby focusing more on managing symptoms, treatments, and the physical and psychosocial consequences of the condition [20]. Although both empowerment and self-control link to gaining control over decisions and actions, empowerment can be considered either a social, cultural, psychological or political process [19]. Whereas self-control seems more an internal process, with self-control being defined as ‘the capacity for altering one’s own responses, especially to bring them into line with standards such as ideals, values, morals, and social expectations, and to support the pursuit of long-term goals’ (p.351) [21]. This also relates to someone’s ability to adapt to new situations [22, 23].

Proceeding from the new definition of health from Huber, ‘having the ability to adapt and self-manage’ (p. 2) [24], having higher levels of self-control at work and the possibility of exerting self-control can improve wellbeing and health. This then facilitates sustainable employment for workers with a chronic condition. Encouraging people with a chronic condition to take control over their lives and their work has been a focal point of the Dutch government and society for a long time [25]. In a qualitative synthesis on self-control of workers with a chronic condition, we specified the desired behaviors that are important to staying at work and the influence of these behaviors and their interaction with the environment. The study findings also showed the importance of support for exerting self-control [26].

Exerting desired self-control behaviors is often not enough to stay at work for workers with a chronic condition. Adequate support is also critical, as stated by the European Chronic Disease Alliance [5]. Research has already shown the positive effects of a supportive work environments for workers with a chronic condition [27]. However, other domains, such as occupational health services and medical health care have an important supportive role as well [28, 29]. National policies can also have an important influence on sustainable employment of workers with a chronic condition [5]. Effective supportive interventions can help workers with a chronic condition to stay at work and achieve sustainable employment. However, workers’ specific support needs must first be identified. Workers with a chronic condition who are successful in staying at work can provide valuable input for these supportive interventions. Therefore, the aim of this study is to explore the lived experiences of Dutch workers with a chronic condition who are successful in staying at work, and identify facilitating factors, existing barriers and possible support needs for staying at work.

## Methods

### Study design

Four focus groups were conducted between August and December 2017 with various types of workers with a chronic condition. This qualitative research method was used to gain an in-depth understanding of how workers with a chronic condition successfully stay at work, the barriers they face in their working life and their need for support. The consolidated criteria for reporting qualitative research (COREQ) were taken into account with the study design and reporting [30]. Representative quotes from the focus groups were translated by a native English speaker and added to illustrate the findings.

### Recruitment

For the recruitment of participants, several Dutch patient organizations (for patients with physical as well as psychological conditions) were approached with the request to promote the study, by placing information about participation on their website, weekly or monthly newsletter or Facebook page. This information included a brief description of the study and a link to the study’s website with more extensive information on the focus groups, inclusion criteria and ways to sign up (registration form, or an e-mail or phone call to the researcher). This brief description of the study and the link to the study’s website was also placed in a call on LinkedIn and the Amsterdam University Medical Center (Amsterdam UMC) website. To recruit self-employed workers, an association that represents the interests of Dutch self-employed workers was contacted and a request was made to promote the study on their LinkedIn community. Participants were eligible for participating in a focus group if they met the following criteria: 1) had a physical or psychological chronic condition; 2) had paid work; 3) were over 18 years of age; 4) had proficiency of the Dutch language. Over 100 workers with one or more physical chronic conditions indicated their willingness to participate in a focus group. To create heterogeneous focus groups, participants were selected through purposeful sampling, taking gender, age, chronic condition, and type of paid work into account. Participants were contacted by email with a detailed description of the study and a suggestion for date and time. If participants had additional questions, the researcher (AB) contacted them by telephone for further clarification. Out of 61 eligible participants who were contacted, 30 actually participated in the focus groups. Reasons for not participating were: 1) not being able to attend at the suggested date and time or 2) having symptom aggravation.

### Participants

Four focus groups were conducted with 30 participants in total. The first three focus groups included employees and (partially) self-employed workers (*n* = 26), and the fourth focus group had only self-employed workers (*n* = 4). Almost half of the participants worked less than 30 h a week, with a minimum of 8 h per week. Most of these participants worked between 20 and 30 h a week. Participants suffered from metabolic conditions, lung conditions, musculoskeletal conditions, neurological conditions, digestive tract conditions or a combination of these. Other participant characteristics are shown in Table 1.

**Table 1:** Characteristics of study participants (*n* = 30)

Characteristics	Number
Sex	
Male	6
Female	24
Age (years)	
Mean	46.6
Range	23–73
Type of employment	
Employee	19
(Partially) self-employed worker	11
Working hours	
< 30	16
> 30	14

### Focus groups

Focus groups were held at the Amsterdam UMC in Amsterdam, the Netherlands. Each focus group lasted approximately 2 h and was conducted in Dutch. The focus groups were moderated by researchers from the research team. An observer was present to assist the moderator and monitor the group interaction. The main researcher (AB), a female health scientist with experience in qualitative research was present at all focus groups, either as a moderator or an observer. During all four focus groups, a secretary was present to take notes. A script with topics and open questions was developed to aid the moderators and ensure comparability of the focus groups, thereby increasing reliability. The open questions were pilot tested with three workers with a chronic condition, one of whom was a participant who was not able to attend a focus group. The other two workers were recruited from the researcher’s own network. The focus groups started by the researcher explaining the study aim and informed consent forms were then signed. The first part of the focus group entailed discussing the participants’ experiences working with a chronic condition, while the second part focused on their perceived support needs to stay at work. An assignment on ‘creating the ideal supporter’ was part of the focus group discussion. At the end of each focus group, participants received a gift certificate and travel expenses were accounted for. Data saturation was achieved after the four focus groups.

### Data analysis

All focus groups were digitally recorded and the data was transcribed verbatim. Transcription was performed by a specialized external agency. Summaries of the focus groups were made and sent to all participants for a member check. Thematic analysis was used to analyze the collected data [31]. The analytic process included several stages starting with reading and rereading of the transcripts to become familiar with the data. An inductive approach was used to analyze the data starting with line-by-line coding of the transcripts. During this open coding process, qualitative data indexing software (ATLAS.ti) was used to assist the coding process and helped to produce an initial list with codes. The next stage of analysis was sifting through the data to search for similarities and discrepancies, and ultimately grouping and combining codes into subthemes in an iterative manner. All data was coded by the main researcher (AB) and by two trained research assistants (health sciences interns). Weekly meetings were held to discuss disagreements in the coding and grouping process until consensus was reached. The last stage consisted of discussions among members of the research team (AB, CB, FS, JA) until consensus was reached on the final themes.

### Ethical considerations

All participants signed an informed consent form at the start of the focus group. Written and oral information was provided to all participants on the confidentiality and anonymity of the results of the study. The Medical Ethical Committee confirmed that ethical approval was not required because The Medical Research Involving Human Subjects Act (‘Wet Medisch-wetenschappelijk Onderzoek met Mensen’) does not apply to this study.

## Results

Themes were identified after data analysis of the lived experiences of workers with a chronic condition. The themes related to personal and environmental factors that helped workers with a chronic condition to stay at work (disclosure, communication and expression of one’s needs, decision-making based on what is important in life, perseverance and securing boundaries, and environmental facilitators). Themes also included remaining barriers in various contexts (knowledge and regulations in the workplace, occupational and medical health services, and social security) and the needs for support to stay at work for workers with a chronic condition (support available to all workers, characteristics of the ideal supporter, and how and when to offer support). An overview of themes is presented in Table 2.

**Table 2:** Overview of themes

Personal and Environmental facilitators	Disclosure
	Communication and expression of one’s needs
	Decision-making based on what is important in life
	Perseverance and securing boundaries
	Environmental facilitators
Environmental barriers	Knowledge and regulations in the workplace
	Occupational and medical health services
	Social security
Support needs	Support available to all workers
	Characteristics of the ideal supporter
	How and when to offer support

### Disclosure

Many participants had disclosed their condition to their employer and co-workers. They determined the right moment to disclose their condition; as long as the participants were able to function at work and the condition was not visible, they often did not feel the urge to disclose their condition. Disclosure brought these workers understanding and support, making it possible for their employers to consider their condition and create work accommodations. One participant explained his sense of relief after having disclosed his condition to his employer, and thus making it possible to be himself at work again. Some participants just felt that disclosing their condition was the right thing to do, since their condition is part of who they are.

*“In solidarity with my colleagues, with whom I always have a very involved relationship, I had something like this: “this is going on in my life, so just as I tell you what I do over the weekend, I also tell you about this (the chronic condition)”.” (FG1, employee)*

Some participants had not disclosed their condition to their employers. Just having started a new job, or fear of losing their jobs were mentioned as reasons for non-disclosure. One participant explained that she kept getting better at making excuses for her inability to perform certain tasks and even wondered herself why she did not disclose her condition.

### Communication and expressing one’s needs

Many participants stated the importance of clear communication and expression of their support needs. Several participants had requested support (e.g., work accommodations) from employers and co-workers when needed. Some participants struggled with the dilemma of asking for help or doing a task themselves. Requesting support did not come naturally for all participants, it sometimes took a while to cross this threshold as expressed by this participant:

*“One day you are able to do it (certain tasks), while the other day….But that really means, and that is a victory I have had to achieve for myself, is that I have to ask. If I do not succeed, then I must say it, because someone else […] no one sees that I am sick.” (FG1, employee)*

### Decision-making based on what is important in life

Many participants expressed their motivation to work and had the desire to stay at work. They spoke extensively about the meaning of work as a facilitator (e.g. financial security, social contacts, participating in society) and the importance of work in relation to other life domains. The right balance between work health and personal life was important for staying at work. This meant having a job that matched their capacities. In addition, participants also had to make decisions based on what they thought was important in life and on how they wished to spend their energy. For some participants, this meant not going to a party or other social event. Others stated that their personal life had become even more important after their diagnosis. Therefore, some had chosen to work fewer hours a week or in a less demanding job, thereby saving enough energy for other activities. One participant explained that she sometimes made a conscious decision to participate in a certain activity for the sake of her mental wellbeing, knowing that she would suffer the consequences later on:

*“Sometimes, I make a conscious decision to go beyond my physical limits because I know I will feel a lot better mentally. I prefer to lie down on the couch the next day like a dead bird and having enjoyed something that I really wanted to do, than be at home and feeling physically okay and think, shit now, I am not part of that (social activity)…” (FG3, employee)*

### Perseverance and securing boundaries

Several participants pointed out that they want to be positive, stay busy and do not like to complain despite their pain or other symptoms. This perseverance helped them to stay at work.

*“I have been in a Facebook group of the Rheumatism Fund and there were only people complaining, “Oh, I have such pain today, yes, so do I”. You know, I got out, I was in it for two days, and I was completely mad. I don't want to be negative, I want to be positive, I want to move forward, I want to look the other way.” (FG1, employee)*

At the same time, guarding boundaries was another crucial facilitator for workers to stay at work. Many participants expressed their difficulty with maintaining boundaries and saying “no” if their workload was too great. Some self-employed workers pointed out that they could decide for themselves how much work to take on. This helped them find the right balance between work, health and personal life. This flexibility was considered to be the great advantage of being self-employed. However, for most self-employed participants, this flexibility was not the reason for becoming self-employed, since the majority had already been self-employed prior to their diagnosis. Having a partner as main breadwinner facilitated this flexibility.

*“You know, and now I can just say “no, I can't do it next week”, while I have a very empty agenda, so to speak, but I, there is no one checking up on me.” (FG4, self-employed worker)*

### Environmental facilitators

Participants considered the support they received from multiple directions as an important facilitator. A partner who helps with household chores and provides emotional support, and an employer who looks after their employees by thinking about solutions to work-related problems were mentioned as important for staying at work. One participant explained how his employer paid for a stay in a spa in Montenegro after his health insurance no longer covered these expenses. Several participants indicated the relevance of recognition by managers and supervisors. One participant illustrated this by describing that her employer paid for her new education, which enabled her to switch to a suitable job within the organization.

*“They themselves have looked at what is useful to do within the organization, what is needed, the study is also paid, that is really ideal.” (FG3, employee)*

Some participants mentioned patient organizations or other groups that provide practical information and support. One participant described the availability of such an organization located in the hospital:

*“I always saw it as a kind of tourist office, they had all sorts of information, they had contact with physiotherapists, they knew everything about high / low desks, rolling stools that were good for people with Bechterew's disease, they had all sorts of information and they were indeed constantly calling out “do you need something, are you doing well at work?”” ( FG3, partially self-employed worker)*

### Knowledge and regulations in the workplace

Many participants spoke about their colleagues and managers’ lack of knowledge on working with a chronic condition. Although not intentional, this lack of knowledge led to unpleasant situations in some cases:

*“Because my supervisor, who thought for me: “I will make the decision for her whether she is allowed to do this or that or that. Or being capable of.” And that is of course, without any consultation, a painful matter. And of course, very frustrating.” (FG2, employee)*

This lack of knowledge coincided with prejudices about certain chronic conditions and a worker’s ability to perform with their condition. A possible result was patronizing or a permanent take-over of certain tasks by co-workers. Some participants explained that this presented a barrier for disclosure or requesting support.

*“But, I am not so fond of patronizing, it is not meant to be wrong, it is only: “Oh, how are you now”? Well, it makes me itch when I think about it and that's why I have sometimes said: “Did I do right to disclose”?” (FG1, employee)*

Participants also mentioned their struggle with rules and regulations within their organizations and how these were applied by managers or supervisors. Several participants mentioned that in some cases regulations were applied at random and not in a fair way.

*“Organizations have their own rules, which are then applied randomly.” (FG2, employee)*

### Occupational and medical health services

Some participants expressed their dissatisfaction with the current guidance and support from their occupational physicians. This was given as a reason for not seeking additional support from these professionals. Being on the side of the employer, a lack of knowledge of chronic conditions or not giving useful advice were underlying causes for this negative attitude toward occupational physicians.

*“And then at some point he did have a tip, the think-along tip, so I work three days, right, 20 hours. Yes, then I could divide those 20 hours over 5 days. Well, I didn't think that was such a good tip.” (FG2, employee)*

A major barrier brought up by a large number of participants was the health care professionals and medical specialists’ lack of attention to employment and paid work during the course of treatment, despite the importance of work for these workers.

*“It would also be nice if a specialist already offered this (information on working with a chronic condition), because working is just very important for everyone. I think that this subject (work) is underexposed, also by the hospital itself, but that is my experience.” (FG2, employee)*

In some cases, this led to advice by medical specialists to quit working or at least reduce working hours:

*“I do not cooperate with him (the specialist). He really finds it amazing that I am still working. He says: “Then reduce (in working hours) a little, then reduce a little”.” (FG2, employee)*

All participants had to deal with doctor or hospital appointments and visits to other health care professionals, such as a physical therapists. These appointments had a significant impact on their work, because in most cases they were forced to plan their appointments during working hours. According to participants, it would be helpful if these appointments could be made in more suitable hours, thereby lessening the impact on their work.

*“The physical therapist for people with rheumatism is available on Tuesday afternoon at one o'clock and Friday afternoon at one o'clock. And if you work all day, then one o’clock is a terrible time […] then you have to exercise for an hour, get stressed out back to work and then you actually still have to work. But you actually don't have the energy anymore to work. So, you know, it (the consultation with physical therapists) just has to be offered in the evening too.” (FG1, employee)*

### Social security

Several of the participants identified the complexity of the Dutch Social Security Institute (DSSI) as a barrier. They explained that they had received or still receive a (partial) benefit from the DSSI. The DSSI enforces many rules, sometimes even contradictory, which makes it a complex, hard-to-understand system as expressed by some participants. This has also led to much distrust towards the DSSI. One participant illustrated her feeling of being thwarted by the DSSI instead of helped:

*“I am still able to work, I still work now. So I can work and then you get advice: “why don't you do volunteer work?” And now, I get the advice: “You have to work fewer hours because otherwise we will give you a fine and we have to reclaim your benefit and everything.” So, next month, I'm going to work fewer hours, but only on paper. So I am actually going to work my own hours, sort of like doing volunteer work in my own job or something.” (FG1, employee)*

None of the self-employed workers had occupational disability insurance. The difficulty of finding insurance with an already existing health problem and the size of the premium served as barriers for obtaining this insurance for self-employed participants. The absence of this financial safety net created feelings of insecurity. Additionally, the self-employed participants spoke about their difficulties with receiving support; they have no one to turn to compared to employees who can ask for help from their employer or occupational health professional. Some self-employed participants explained that they sometimes ask their physical therapist, specialist nurse or friends for advice on how to cope with certain problems.

*“So, there is no contact person, there is no one focused on self-employed entrepreneurs, and therefore there is absolutely no help, you must have had very good assertiveness training first before you can get any help at all.” (FG4, self-employed worker)*

One participant spoke of the lack of information provided by the DSSI. She knew the DSSI could offer some support, however, when she contacted them, no one could help her.

*“But for work things you actually have nothing. But it seems, although there is nothing on writing, but the DSSI can certainly help you make your work easier. […]But there is nobody (at the DSSI) available. If you call the DSSI nobody knows anything.” (FG4, self-employed worker)*

### Support available to all workers

Participants pointed out that support should be made available to all workers, employees as well as self-employed workers, even for people with a chronic condition who would like to enter the labor market. By paying more attention to paid work in the treatment processes of chronic conditions, support can be made available to all these workers. Some participants suggested an occupational physician at the outpatient clinic, since this would make support available for all who need it.

*In my ideal situation, there is a company doctor at an outpatient clinic where you don't have the hassle that a medical specialist says A and a company doctor says B, but that together they eh. And then, the company doctor is also accessible for people like us who are self-employed, but also for people who are looking for a job. They (people looking for work) also do not have a company doctor.” (FG2, self-employed worker)*

More support for self-employed workers must also be made available, as illustrated by one participant describing her search for someone who could offer support:

*“Yes, I am very much looking for someone who can help me. And I am also wandering in the desert of a rehabilitation doctor, company doctor, ‘Heliomare’ (a rehabilitation clinic), and what else…” (FG4, self-employed worker)*

### Characteristics of the ideal supporter

Support should meet certain criteria as expressed by all participants. Support must be easily accessible, based on equivalence and the supporter must assume the possibilities of the worker. Other participants added the importance of the supporter having a proactive and personal approach, and considering the person’s work as well as their personal situation and mental wellbeing. One participant pointed out that support should be based on workers’ motives to work.

*“But, say in the guidance there must be an eye on one’s motivation. For some, the motivation to work is pure money, so okay, […] but how are we going to ensure that I have my money at the end of the month? […] now you are now being thwarted in working while your motivation is in the work itself. So I think there should also be an eye on that.” (FG1, employee)*

Several participants pointed out that the person offering support should serve as a coach or sparring partner. A wide variety of supporters were mentioned when asked who is the most suitable person to take on this supporting task: specialized nurses, social workers, occupational therapists, independent advisors, experienced experts or occupational physicians. Occupational physicians were often mentioned by participants in paid employment, since they could play a bridging role between the employee, employer and medical specialist.

### How and when to offer support

Several participants indicated that support should be set up prior to the start of problems. Another participant mentioned that support should be offered throughout a working career. The majority of participants pointed out the importance of customizing support, since every worker has his or her own needs.

*It's about searching for that piece of customization, you would want someone who makes a tailor-made suit for you, I say.”(FG3, self-employed worker)*

Participants spoke about the various areas where support is needed. It became clear that information is needed on the rights and obligations of employees and employers with regard to sick leave and social security. Practical advice on work accommodations and job coaching were also mentioned as areas for support. Some participants spoke about the need for a sympathetic ear whenever they just want to talk.

*“Of course, there is always a bit of emotion added, we all have bad moments, that we are at the end of our rope, for whatever reason. I need a listening ear and understanding, recognition.” (FG4, self-employed worker)*

## Discussion

This study described the lived experiences of workers with a chronic condition, who were successful in staying at work. Facilitating factors were identified in the personal and environmental domains. Disclosure, being clear about one’s needs, knowing what is important in life and making subsequent decisions are important to staying at work. Environmental support (e.g. social and employer support) was an important facilitator. Despite the fact that these workers were able to stay at work with their condition, barriers still remained. Barriers in the work environment, the health care system and with the national occupational health and social security services included: lack of knowledge, lack of a clear policy and compliance to regulations in the work environment, dissatisfaction with occupational physicians’ support, lack of focus on work in the course of treatment of chronic conditions, the complex system of the DSSI and the absence of a financial safety net for self-employed entrepreneurs. The need for support to facilitate workers staying at work also included support being available for employees and self-employed entrepreneurs, proactive support and support customized to a worker’s individual needs.

### Comparison with the literature

Multiple models on work and work disability demonstrate the complex system of sustainable employment for workers with a chronic condition and the various stakeholders involved. It becomes clear that workers with a chronic condition have to deal with many people in multiple domains, e.g. medical specialists and nurses in the health care system, employers and co-workers in the work environment, occupational health professionals, and family and friends in the social environment [32]. The self-control model we developed in our qualitative synthesis illustrates the behaviors that can help with staying at work, and the influence of various contexts on behavioral expression. The facilitating factors identified in this study correspond in a large part with the behaviors in our model, e.g. disclosure, requesting accommodations and support, and finding a healthy balance [26]. Moreover, these facilitating factors are also in line with several motivators (e.g. meaning of work) and success factors (e.g. perseverance) for staying at work, as described in a qualitative study by De Vries et al. [33]. Additionally, the identified barriers make it clear that it is not just the work environment that is important to staying at work, but also occupational and medical health services and social security services are relevant.

The importance of support for self-employed workers with a chronic condition was identified in a study by Adam et al. [34]. Financial, practical as well as emotional support are all relevant [35, 36]. Self-employed workers lack access to support in contrast to employees who can turn to their employer, occupational physician or other occupational health care professional [37]. The Netherlands is not the only country with this problem for self-employed workers. A study by Torp et al. conducted in Norway also described this and the subsequent need for a network of professional support for self-employed entrepreneurs [35]. Providing occupational health services in hospital outpatient clinics might be a good addition to the existing Dutch care models, and make services more available to all workers.

A study by Mittag et al. comparing social security in the Netherlands, Finland and Germany, pointed out that ‘structured, close communication’ between stakeholders is a facilitator for a successful return to work. According to the article, the DSSI is committed to coordinated and structured practices (p. 1087) [38]. The participants’ views on DSSI’s complexity and lack of support makes us suspect that this does not always work out as planned. The lack of information provision, as indicated by some self-employed workers in our study, was also addressed by other self-employed workers with a chronic condition. In a group discussion with a number of self-employed members from several patient organizations, the respondents explained that vital information on starting entrepreneurship was not promoted by the DSSI [36].

### Strengths and limitations

This study illustrates the success stories of both employees and self-employed workers with a chronic condition who are still in paid work, the barriers that still remain and subsequent support needs. However, some limitations to this study have to be mentioned. Although attempts were made to create heterogeneous groups of participants, we did not fully succeeded in this. A large proportion of participants suffered from specific physical chronic conditions, such as rheumatism and multiple sclerosis. None of the participants had a psychological disorder, despite the efforts to recruit workers with psychological disorders. This may be a result of the eloquence of these specific groups, in contrast to workers with mental illnesses, for example [39, 40]. Adding focus groups with workers with psychological disorders might have revealed other barriers or support needs, such as dealing with stigmatization. Although disclosure was an important aspect among the participants of this study, research conducted on the various barriers for workers with psychological disorders, shows that stigmatization and discrimination after disclosure is a particular problem for this group of workers [41–43]. A study by Brouwers et al. also shows the different factors that influence the outcome of disclosure, e.g. workplace, financial and employee factors [43]. Second, none of the participants worked in a profession requiring heavy physical labor. Sustainable employment in jobs with a heavy physical workload is even more challenging. Therefore, the absence of workers in these physical demanding jobs in this study, may be caused by the fact that workers with a chronic condition are less likely to work in these kind of professions since they often have already switched to less physically demanding jobs to continue working or end up taking sick leave. Third, only a small proportion of participants in the focus groups were male. Only a small number of men signed up in comparison to the number of women. All men were contacted for participation in a focus group. A possible explanation could be that women might be more willing to disclose their condition and talk about their situation in a focus group than men [44].

### Implications for practice, policy and research

Staying at work and preventing work-related problems at an early stage are important to workers with a chronic condition. The work environment, the health care system and national regulations and services can guide and support workers to stay at work. Barriers and support needs identified in this study have implications for policy, practice and research. Interventions aimed at eliminating environmental barriers, e.g., training employers, optimizing occupational health services and integrating work into medical health care could have a large impact on sustainable employment, and these actions are recommended by European organizations committed to improve work participation for workers with a chronic condition [5, 45].

Receiving support is important to all workers, since participants pointed out that every worker should have access to occupational health services, including self-employed workers. The coverage of occupational health services in the Netherlands (80%) is the percentage of workers with access to these services [46]. Although this number looks promising, it does not provide a complete picture. The fact that workers have access to occupational health services, does not mean they will actually use them, as in the case of participants dissatisfied with the offered support. Additionally, provided support does not always meet workers’ needs. More tailored support should be made available, since every worker has specific needs. A proactive approach with an eye to all domains of life (work and personal situation), as indicated by our participants, was also described in a focus group study by Vooijs et al. [12]. Improving support from medical health care by focusing more on work and employment in the course of treatment, will aid workers with a chronic condition to manage work-related problems at an earlier stage. Failure to discuss work in the healthcare consultation room was identified as a problem several years ago [47]. In addition, collaboration between occupational physicians, medical specialists and employers is critical. Currently, the collaboration between occupational physicians and other medical specialists is suboptimal [48].

The integration of work into the course of treatment is relevant for health care professional training (e.g., medical specialists or specialized nurses). Including work as a theme in health care professional training can help create awareness about the meaning of work for patients. Inter-disciplinary cooperation between occupational physicians and other physicians can possibly be improved by stating shared goals or joint educational programs [48]. Occupational physicians in an outpatient clinic of the hospital makes them better accessible for workers as well as health care professionals for advice on work related problems. Current support by occupational physicians should be more tailored to workers’ support needs and information on policy and regulation should be made available in a clear and understandable way to employees and employers. Last, policy makers in the Netherlands should think about ways to support self-employed workers with professional, practical and financial advice.

More interventions are needed on integrating work during treatment and medical specialists’ decision-making processes. Considerable research has been conducted on employees with a chronic condition in comparison to self-employed workers. Although this study tries to fill this gap, more research is needed on self-employed workers with a chronic condition and the optimization of their support system.

## Conclusions

Personal and environmental facilitators help workers with a chronic condition to successfully stay at work. However, barriers to sustainable employment still remain in the context of their work environment, the health care system and the provision of occupational health and social security services. Support to all workers with a chronic condition, employees and self-employed workers, is needed and should be tailored to the specific needs of the individual worker.

## Data Availability

The datasets used and/or analyzed during the current study are available from the corresponding author by reasonable request.

## Abbreviation

DSSI: Dutch Social Security Institute
